# Spin-splitting effects on the interband optical conductivity and activity of phosphorene

**DOI:** 10.1038/s41598-020-65951-9

**Published:** 2020-06-08

**Authors:** Le Thi Thu Phuong, Tran C. Phong, Mohsen Yarmohammadi

**Affiliations:** 1 Center for Theoretical and Computational Physics, University of Education, Hue University, Hue, Viet Nam; 2 The Vietnam National Institute of Educational Sciences, 101 Tran Hung Dao, Hanoi, Viet Nam; 3 Department of Energy Engineering and Physics, Amirkabir University of Technology, 14588 Tehran, Iran

**Keywords:** Optical materials and structures, Two-dimensional materials

## Abstract

Being able to tune the anisotropic interband transitions in phosphorene at finite temperature offers an enormous amount of possibilities in finding new insights in the optoelectronic community. To contribute to this goal we propose a Zeeman spin-splitting field aiming at absorbing various frequencies of the incident light. Employing the tight-binding Hamiltonian to describe the carrier dynamics and the Kubo formalism to formulate the orientation-dependent interband optical conductivity (IOC) and optical activity of phosphorene we investigate the absorption and scattering mechanisms in phosphorene depending on the Zeeman field strength and optical energy parameters. The optical activity features are characterized by exploring the eccentricity and shift phase of reflected and transmitted electromagnetic waves of the incident light. Different electronic phases in the absence and presence of Zeeman field ultimate different types of interband transitions of which in all cases the IOC along the armchair direction is larger than the zigzag one. However, we observed an irregular (regular) process for IOC with the Zeeman field along the armchair (zigzag) direction, resulting in irregular (regular) absorption and scattering mechanisms. Additionally, a little to no effects for temperature-dependent IOC are provided with the Zeeman field in undoped phosphorene. Further, almost linearly and elliptically polarizations are reported for the transmitted and reflected waves, respectively, indicating that the phosphorene is almost transparent. The emergence of Zeeman spin-splitting effects in optoelectronic properties of phosphorene is pleasant to make it a great potential candidate for logic applications.

## Introduction

The substantial amount of interest in layered two-dimensional (2D) group-IV and -V materials has fascinated immense experimental and theoretical researchers in exfoliating and/or fabricating other 2D materials^1–9^. In a nutshell, graphene possess a zero band gap^10,11^, while silicene, transition-metal dichalcogenides and germanene have a finite band gap in their structure^12–15^. On the one hand, while gapped 2D structures illustrate a high on/off current ratio, graphene suffers from a low ratio^16,17^. On the other hand, the charge carrier mobility in gapped 2D materials is much lower than graphene. These imply that there are some restrictions on their traditional electronic and optoelectronic applications. These deficiencies pushed experimentalists to synthesize new 2D systems with high enough on/off ratios and carrier mobilities. One of more than 600 stable exfoliated 2D lattices^12^ is 2D black phosphorus (BP)^18–21^. Monolayer BP (MBP) possess a direct finite bandgap of 1.52 eV^22–26^, which decreases when the number of layers is increased^22,23,27,28^. Interestingly, BP owns an on/off ratio and carrier mobility of about 10^5^ and 10^3^ cm^2^/Vs, respectively^19^.

Arising from the $s{p}^{3}$-hybridization of $3s$, $3{p}_{x}$, $3{p}_{y}$, and $3{p}_{z}$ orbitals of phosphorus atoms (_15_P: $1{s}^{2}$ $2{s}^{2}$ $2{p}^{6}$ $3{s}^{2}$ $3{p}^{3}$) the crystal structure of MBP is puckered, which is quite different than honeycomb lattices of graphene, group-IV and -V 2D materials^22^. This discrepancy leads to anisotropic electronic and optoelectronic features, i.e. direction-dependent properties^20,28–35^, resulting in different particle effective masses for two in-plane directions. For instance, the momentum-dependent energy dispersion along the $x$- and $y$-direction, i.e. ${\mathcal E} ({k}_{x})$ and ${\mathcal E} ({k}_{y})$, respectively, is almost linear and parabolic in the vicinity of the Fermi energy ${{\mathcal{E}}}_{{\rm{F}}}$ (See Fig. 2)^22^. One of the advantages of phosphorene with this anisotropic phenomena in comparison with other 2D materials can be revealed in the tuning of thermoelectric applications to achieve high electrical conductivity and low thermal conductivity in different directions when the thermal gradient and potential difference are applied in different directions. In recent years, the physical properties of BP have been extensively studied both theoretically and experimentally^19,22,26,32,33,35–38^. It has been shown that the low-lying electronic band structure of MBP is easily tuned by external perturbations. For example, potassium doping leads to a semiconductor-to-band-inverted semimetal phase transition with linear ($x$-direction) and parabolic ($y$-direction) dispersions^31,39^ at a critical doping. Moreover, it has been identified experimentally that a semiconductor-to-semimetal transition occurs in BP when hydrostatic pressure is applied^40^. Or a uniform perpendicular electric field originating from gate voltage results in an increase of the band gap^41^. Also, an anisotropic electronic phase transition has been reported in MBP in the presence of dilute charged impurity^42^. Besides the electronic properties, due to the inherent highly dispersive electronic band structure of MBP, a large optical conductivity has been obtained in MBP^25^ of which in the absorption spectra the absorption peaks for the $x$- and $y$- direction take place in different optical frequencies. These, in turn, may avail the realization of polarization sensors and plasmonic devices^33^. In short, BP possesses a maximum optical conductivity along the $x$-direction because it absorbs less light energy polarized along the $x$-direction.

**Figure 1:**
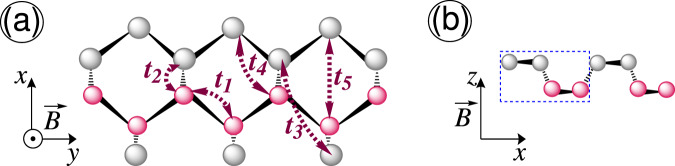
Top (**a**) and side (**b**) view of single-layer phosphorene, respectively. The unit cell of phosphorene with four phosphorus atoms is shown in panel (b) with a blue dashed rectangle. The Zeeman splitting field $\overrightarrow{B}={ {\mathcal B} }_{z}{\hat{e}}_{z}$ is applied to the system perpendicularly, shown by the symbol $\odot$ in (**a**).

**Figure 2:**
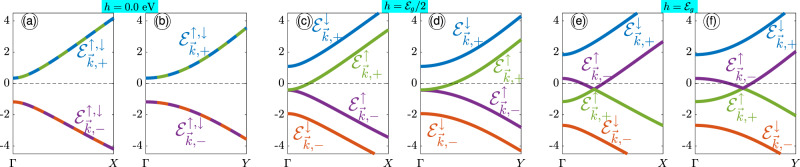
Spin-splitting effects on the electronic band structure of single-layer phosphorene along both armchair and zigzag edges at {(**a**,**b**)} $h=0$ eV, {(**c**,**d**)} $h={{\mathcal{E}}}_{g}/2$ and {(**e**,**f**)} $h={{\mathcal{E}}}_{g}$. The black dashed horizontal lines at ${\mathcal{E}}=0$ eV is the Fermi energy level (${{\mathcal{E}}}_{{\rm{F}}}=0$ eV).

A great amount of faith in finding appropriate candidates for the spintronic industry is put into the condensed matter systems. In ref. ^43^, the researchers have theoretically studied the linear dichroism and the Faraday rotation of strained few-layer phosphorene, where strain is applied uniaxially along the armchair or zigzag direction of the phosphorene lattice. Using the Kubo formula within the tight-binding approach, it has been shown that the linear dichroism and the Faraday rotation of few-layer phosphorene can be significantly modulated by the applied strain. Additionally, using a self-consistent tight-binding approach combined with the standard Kubo formula, it has been reported that the optical conductivity and the linear dichroism of few-layer phosphorene can be modulated by a perpendicular electric field^44^. Alongside the vast amount of works on electronic perturbation effects in phosphorene, there come a small number of theoretical concepts trying to describe *magnetic* perturbation effects to help to improve associated problems in spintronics. To this end, over the years of research, a few works with different purposes have been investigated^45–49^. In ref. ^45^, the dependency of Landau levels on the magnetic field linearly was found. In 2015, Tahir *et al*.^46^ derived the band structure of phosphorene and discussed when a magnetic field is applied perpendicularly, leading to significant anisotropic magneto-optical properties. Similar calculations have been performed on a 2D electron gas in phosphorene multilayer depending on the applied perpendicular magnetic field, resulting in quadratic polynomial-dependentLandau level index^47^. In refs. ^48,49^, the Landau levels have been investigated in MBP, bilayer BP and BP thin films in order to find the role of a perpendicular magnetic field in magneto-electronic and -optical transport properties. Another theoretical work^36^ on magnetic quantization effects in phosphorene was devoted to a comprehensive understanding of a composite magnetic and electric field impacts. Moreover, in the work of Mogulkoc *et al*.^50^ the noticeable deviations of Landau levels from the linear dependence on the magnetic field in phosphorene have been studied. From the works abovementioned, it is easy to find that many efforts have been made to understand the dynamics of charge carriers in phosphorene under magnetic perturbations. Accordingly, to realize novel optical properties in phosphorene, to the best of our knowledge, the role of Zeeman spin-splitting is not well reported theoretically so far, which is a problem of practical importance as it determines observable spintronic transport features.

In the present work, we directly use the tight-binding Hamiltonian of MBP^22,23,35,51^ in the presence of the Zeeman field to study the spin-dependent electronic and optical properties. We will focus on the large enough incident optical energy regime of which charge carriers absorb this energy and transmit from the valence band to the conduction band. We use the linear response theory associated with the Kubo formula including the energy dispersions and electronic states to evaluate both real and imaginary parts of the interband optical conductivity of MBP along the armchair and zigzag directions. Simultaneously, the optical activity of MBP in the presence of an incident perpendicular circularly right-handed polarized light is investigated when the Zeeman field strength is weak and/or strong.

The rest of this paper is structured as follows. We present a short overview of the proper tight-binding Hamiltonian model of MBP in Sec. 2. In Sec. 3 we derive the interband optical conductivity based on the eigenvalues and eigenfunctions obtained from the Hamiltonian by using the Kubo formalism. The model for the optical activity of MBP when propagating electromagnetic waves through phosphorene is given in Sec. 4. We proceed with a discussion of the numerical results in the long-wavelength limit for incident light in Sec. 5. Finally, we briefly conclude the remarks and summarize the results in Sec. 6.

## Theoretical model

In this section, we intend to provide the tight-binding Hamiltonian model based on the atomic configuration of phosphorus (P) atoms in monolayer phosphorene consisting of four P atoms and two sublayers, as shown in Fig. 1(a,b). Thanking the refs. ^22,23,35^ for the effective Hamiltonian of phosphorene in the presence of the Zeeman splitting field in real space, the total Hamiltonian ${\mathcal{H}}$ is composed of two different parts of which the key purpose is to gain control over the spins:

1 $${\mathcal{H}}=\sum _{\langle i,j\rangle ,s}\,{t}_{ij}{\hat{f}}_{i,s}^{\dagger }{\hat{f}}_{j,s}+h\,\sum _{i}\,{\hat{f}}_{i,\uparrow }^{\dagger }{\hat{f}}_{i,\downarrow }+{\rm{H.c}}\mathrm{.,}$$

where the summation runs over all nearest neighbor lattice sites up to fifth neighbors and the symbol $\langle i,j\rangle$ means that $i\ne j$. Also, the summation over $s$ refers to the spin of electrons, i.e. $s=\uparrow ,\,\downarrow$. Note that the effect of perpendicular magnetic field on the orbital motion of charge carriers needs a correction to all hopping parameters with Peierls substitution, which has been addressed already^52–54^. However, the spin contributions are still missing in the literature and we restrict ourselves to the case of spin-splitting only in the present paper. It is necessary to mention that the magneto-optical properties of phosphorene due to the Peierls contribution have been addressed as well, e.g. in ref. ^55^ the electronic and magneto-optical properties of rectangular, hexangular, and triangular monolayer phosphorene quantum dots (MPQDs) utilizing the tight-binding method are fully investigated. This means that including the effect of perpendicular magnetic field on the orbitals does not show new physical insights related to the present paper, while it does on the spin-splitting. So, the coefficient $h=g{\mu }_{B}{{\mathcal{B}}}_{z}$ stands for the applied Zeeman magnetic field potential, where $g$ is the Lande factor, ${\mu }_{B}$ is the Bohr’s magneton and ${{\mathcal{B}}}_{z}$ is the perpendicular magnetic field strength. Having Zeeman term here we can further manipulate MBP which presents us with great potential to create spintronic devices of which the number of applications is immense. We envisage the operators ${\hat{f}}_{i,s}^{\dagger }$ and ${\hat{f}}_{j,s}$ to create and annihilate an electron at *i*-th and *j*-th atomic site with spin $s$, respectively. The hopping integral energy ${t}_{ij}$ is for the hopping between atomic sites $i$ and $j$, which are given by ${t}_{1}=-\,1.220$ eV, ${t}_{2}=+\,3.665$ eV, ${t}_{3}=-\,0.205$ eV, ${t}_{4}=-\,0.105$ eV, and ${t}_{5}=-\,0.055$ eV^22,35^, as drawn in Fig. 1(a).

By considering the same atoms for both sublayers, the momentum-dependent Hamiltonian for one-orbital *p*_*z*_–like the tight-binding model in the low-energy limit after the Fourier transformation reads^35^

2 $${\mathcal{H}}=\sum _{\overrightarrow{k}}\,{\psi }_{\overrightarrow{k}}^{\dagger }{{\mathcal{H}}}_{\overrightarrow{k}}{\psi }_{\overrightarrow{k}},$$

where the momenta $\overrightarrow{k}=({k}_{x},{k}_{y})$ belong to the first Brillouin zone (FBZ) of phosphorene and the wave function is defined by ${\psi }_{\overrightarrow{k}}^{\dagger }=\{{a}_{\overrightarrow{k}}^{\dagger ,\uparrow },{b}_{\overrightarrow{k}}^{\dagger ,\downarrow },{a}_{\overrightarrow{k}}^{\dagger ,\uparrow },{b}_{\overrightarrow{k}}^{\dagger ,\downarrow }\}$ with ${{\mathcal{H}}}_{\overrightarrow{k}}$^51^

3 $${{\mathcal{H}}}_{\overrightarrow{k}}=(\begin{array}{cccc}{f}_{\overrightarrow{k}} & h & {g}_{\overrightarrow{k}} & 0\\ h & {f}_{\overrightarrow{k}} & 0 & {g}_{\overrightarrow{k}}\\ {g}_{\overrightarrow{k}}^{\ast } & 0 & {f}_{\overrightarrow{k}} & h\\ 0 & {g}_{\overrightarrow{k}}^{\ast } & h & {f}_{\overrightarrow{k}}\end{array}).$$

The structure factor elements of the Hamiltonian above, i.e. momentum-dependent terms, are given by

4a $${f}_{\overrightarrow{k}}=4{t}_{4}\,\cos \,({k}_{x}a/2)\,\cos \,({k}_{y}b/2),$$

4b $${g}_{\overrightarrow{k}}=2{t}_{1}{e}^{-i{k}_{x}{a}_{1x}}\,\cos \,({k}_{y}b/2)+{t}_{2}{e}^{i{k}_{x}{a}_{2x}}+2{t}_{3}{e}^{i{k}_{x}{a}_{3x}}\,\cos \,({k}_{y}b/2)+{t}_{5}{e}^{-i{k}_{x}{a}_{5x}},$$

in which we have used the symmetry property between atoms in two different sublayers. Parameters ${a}_{\{1,2,3,4,5\}x}$ are the distance between the intra- and interplanar nearest-neighbor atoms projected to the $x$ direction, given by ${a}_{1x}=1.41763$ Å, ${a}_{2x}=2.16400$ Å, ${a}_{3x}=3.01227$ Å, ${a}_{4x}=2.21468$ Å, and ${a}_{5x}=3.63258$ Å for $a=4.42936$ Å (the length of the unit cell into the armchair direction) and $b\mathrm{=3.27}$ Å (the length of the unit cell into the zigzag direction)^22,35,51^. By diagonalizing the Hamiltonian in Eq. (3), the spin-dependent electronic dispersions comprising the conduction (+) and valence (−) bands can easily be calculated as:

5a $${{\mathcal{E}}}_{\overrightarrow{k},+}^{\downarrow }={f}_{\overrightarrow{k}}+\sqrt{{g}_{\overrightarrow{k}}{g}_{\overrightarrow{k}}^{\ast }}+h,\,{{\mathcal{E}}}_{\overrightarrow{k},+}^{\uparrow }={f}_{\overrightarrow{k}}+\sqrt{{g}_{\overrightarrow{k}}{g}_{\overrightarrow{k}}^{\ast }}-h,$$

5b $${{\mathcal{E}}}_{\overrightarrow{k},-}^{\uparrow }={f}_{\overrightarrow{k}}-\sqrt{{g}_{\overrightarrow{k}}{g}_{\overrightarrow{k}}^{\ast }}+h,\,{{\mathcal{E}}}_{\overrightarrow{k},-}^{\downarrow }={f}_{\overrightarrow{k}}-\sqrt{{g}_{\overrightarrow{k}}{g}_{\overrightarrow{k}}^{\ast }}-h.$$

Figure 2 shows the spin-dependent electronic band structures along the high symmetry points $\Gamma -X$ and $\Gamma -Y$ of the FBZ of MBP in the absence and presence of the spin-splitting field. At first glance, the high anisotropy result stemming from the anisotropic carrier Fermi velocities and effective masses along the different directions of phosphorene is confirmed in panels (a) and (b), in good agreement with refs. ^22,23,35,51^. The bandgap is of about ${{\mathcal{E}}}_{g}=1.52$ eV. In panels (a) and (b), one can see easily that the spin degeneracy is evident when the spin-splitting Zeeman field is off. As soon as the Zeeman field is increased, the spin-splitting appears, as represented in panels (c)-(f). To ensure that the Zeeman field has an effect on the excitation we set $h={{\mathcal{E}}}_{g}/2$ and $h={{\mathcal{E}}}_{g}$ because even with magnetic proximity effects, reaching to higher induced magnetic fields is impossible^56^. If both quantities were too far from one another there would be no proper excitation, meaning the system stays in its initial equilibrium state. As usual, the spin-up state is shifted down in energy by $2h$, while the spin-down state is shifted up by the same amount, leading to a gapless phase for spin-up states and a gapped phase for spin-down ones at $h={{\mathcal{E}}}_{g}/2$. However, by increasing the Zeeman field strength further new insights come into play; while a gapped phase appears for spin-down states, besides the band inversion, a Dirac-like cone takes place along both directions from $\Gamma -X$ and $\Gamma -Y$ for spin-up ones. These band changes lead to different interband optical transitions compared to the spin degeneracy case.

## Interband optical conductivity

As most of the relations are textbook knowledge, we will provide some references the following theories are primarily based on. We use the Kubo formula^57–59^ to derive the optical conductivity of monolayer phosphorene to know how monolayer phosphorene responds to an applied optical field $\overrightarrow{E}(\omega )$ in the presence of the Zeeman field. Since the considered system is undoped, thus, we only focus on the interband transitions in hopes of gaining new insights. Very recently, it has been shown by self-consistent tight-binding calculations that when a single top (bottom) gate is applied to phosphorene, the system becomes *n*-type (*p*-type) doped with a finite density of electrons (holes) in the conduction (valence) band^60^. In that case, the carrier concentration contribution comes to play the role and intraband transitions cannot be neglected, however, the charge screening effects due to the gating is out of the scope of the present work. For the non-interacting Hamiltonian in the second quantization representation ${{\mathcal{H}}}_{\overrightarrow{k}}^{0}={\sum }_{\overrightarrow{k},\pm ,s}\,{{\mathcal{E}}}_{\overrightarrow{k},\pm }^{s}{\hat{f}}_{\overrightarrow{k},\pm ,s}^{\dagger }{\hat{f}}_{\overrightarrow{k},\pm ,s}$, the total Hamiltonian including the interacting term in the presence of the optical field is given by

6 $${\mathcal{H}}={{\mathcal{H}}}_{\overrightarrow{k}}^{0}+{{\mathcal{H}}}_{{\rm{int}}},$$

where ${{\mathcal{H}}}_{{\rm{int}}}=\overrightarrow{J}\cdot \overrightarrow{A}$. The vector potential $\overrightarrow{A}$ can be calculated using the relation $\overrightarrow{E}(\omega )=-\,{\partial }_{t}\overrightarrow{A}$. On the other hand, the current density $\overrightarrow{J}$ along the Cartesian direction $\alpha$ in the interacting term is related to the $\beta$ components of the respective electric field ${{\mathcal{E}}}_{\beta }$ within the linear response of the optical conductivity $\sigma$ as

7 $${J}_{\alpha }=\sum _{\beta }\,{\sigma }_{\alpha \beta }{{\mathcal{E}}}_{\beta }.$$

The Greek indices $\alpha$ and $\beta$ may take the values of the flavors $x$ and $y$.

In order to obtain the current density vector $\overrightarrow{J}$, the wave vector in the wave function of the non-interacting Hamiltonian should be converted to $\overrightarrow{k}+(e/\hslash )\overrightarrow{A}$. By this, one yields the *α*-component of $\overrightarrow{J}$ as

8 $${J}_{\alpha }=-\,\frac{e}{\hslash }\,\sum _{\overrightarrow{k},\lambda }\,{\hat{f}}_{\overrightarrow{k},\lambda }^{\dagger }{\hat{f}}_{\overrightarrow{k},\lambda }{\nu }_{\overrightarrow{k}}^{\alpha }+i\frac{e}{\hslash }\,\sum _{\overrightarrow{k},\lambda }\,{\hat{f}}_{\overrightarrow{k},\lambda }^{\dagger }{\hat{f}}_{\overrightarrow{k},-\lambda }{\chi }_{\overrightarrow{k}}^{\alpha },$$

where the first (second) term is known as the paramagnetic (diamagnetic) term and $\{{\nu }_{\overrightarrow{k}}^{\alpha },{\chi }_{\overrightarrow{k}}^{\alpha }\}$ are connected to the velocity of carriers in phosphorene. It is straight forward but tedious to deduce (more sophisticated calculations for the derivation presented here can be found in refs. ^22,23,35,51^)

9a $$\begin{array}{rcl}{\nu }_{\overrightarrow{k}}^{x} & = & +2{t}_{1}{a}_{1x}\,\sin \,({k}_{x}{a}_{1x}+{\theta }_{\overrightarrow{k}})+{t}_{2}{a}_{2x}\,\sin \,({k}_{x}{a}_{2x}-{\theta }_{\overrightarrow{k}})\\  &  & +\,2{t}_{3}{a}_{3x}\,\cos \,({k}_{y}b/2)\,\sin \,({k}_{x}{a}_{3x}-{\theta }_{\overrightarrow{k}})\\  &  & +\,2{t}_{4}a\,\sin \,({k}_{x}a/2)\,\cos \,({k}_{y}b/2)\\  &  & +\,{t}_{5}{a}_{5x}\,\sin \,({k}_{x}{a}_{5x}+{\theta }_{\overrightarrow{k}}),\end{array}$$

9b $$\begin{array}{rcl}{\nu }_{\overrightarrow{k}}^{y} & = & +b{t}_{1}\,\sin \,({k}_{y}b/2)\,\cos \,({k}_{x}{a}_{1x}+{\theta }_{\overrightarrow{k}})\\  &  & +\,b{t}_{3}\,\sin \,({k}_{y}b/2)\,\cos \,({k}_{x}{a}_{3x}+{\theta }_{\overrightarrow{k}})\\  &  & +\,2{t}_{4}b\,\cos \,({k}_{x}a/2)\,\sin \,({k}_{y}b/2),\end{array}$$

9c $$\begin{array}{rcl}{\chi }_{\overrightarrow{k}}^{x} & = & -2{t}_{1}{a}_{1x}\,\cos \,({k}_{y}b/2)\,\cos \,({k}_{x}{a}_{1x}+{\theta }_{\overrightarrow{k}})\\  &  & +\,{t}_{2}{a}_{2x}\,\cos \,({k}_{x}{a}_{2x}-{\theta }_{\overrightarrow{k}})\\  &  & +\,2{t}_{3}{a}_{3x}\,\cos \,({k}_{y}b/2)\,\cos \,({k}_{x}{a}_{3x}-{\theta }_{\overrightarrow{k}})\\  &  & -\,{t}_{5}{a}_{5x}\,\cos \,({k}_{x}{a}_{5x}+{\theta }_{\overrightarrow{k}}),\end{array}$$

9d $${\chi }_{\overrightarrow{k}}^{y}=+\,b{t}_{1}\,\sin \,({k}_{y}b/2)\,\sin \,({k}_{x}{a}_{1x}+{\theta }_{\overrightarrow{k}})-b{t}_{3}\,\sin \,({k}_{y}b/2)\,\sin \,({k}_{x}{a}_{3x}-{\theta }_{\overrightarrow{k}}).$$

The phase ${\theta }_{\overrightarrow{k}}$ is defined as

10 $${e}^{i{\theta }_{\overrightarrow{k}}}=\sqrt{\frac{{g}_{\overrightarrow{k}}}{{g}_{\overrightarrow{k}}^{\ast }}}.$$

Finally, the normalized optical conductivity (with respect to the planar area) within the linear response theory is given by

11 $${\sigma }_{\alpha \beta }(\omega )=\frac{1}{\hslash \omega }\,{\int }_{0}^{\infty }\,dt{e}^{i\omega t}\langle [{J}_{\alpha }(t),{J}_{\beta }(0)]\rangle ,$$

where from the structure symmetry of phosphorene, the contributions of the Hall conductivities are zero, i.e. ${\sigma }_{xy}(\omega )={\sigma }_{yx}(\omega )=0$. From this point, the normalized longitudinal interband optical transitions (IOC) along the armchair and/or zigzag directions are calculated as

12 $$\frac{{\sigma }_{\alpha \alpha }^{{\mathtt{inter}}}(\omega )}{{\sigma }_{0}}=-\,\frac{4i}{\hslash \omega }\,\sum _{\overrightarrow{k}\in {\rm{FBZ}}}\,{({\chi }_{\overrightarrow{k}}^{\alpha })}^{2}\frac{({n}_{\overrightarrow{k},+}^{{\rm{FD}}}-{n}_{\overrightarrow{k},-}^{{\rm{FD}}})({{\mathcal{E}}}_{\overrightarrow{k},+}-{{\mathcal{E}}}_{\overrightarrow{k},-})}{{(\hslash \omega +i\eta )}^{2}-{({{\mathcal{E}}}_{\overrightarrow{k},+}-{{\mathcal{E}}}_{\overrightarrow{k},-})}^{2}},$$

where ${\sigma }_{0}={e}^{2}/\hslash$ and $\eta =10$ meV are the universal value for the optical conductivity and the finite damping between the valence and conduction bands, respectively. The value of $\eta$ is determined phenomenologically and there is no experimental perspective for that. Of course, the artificial relaxation of $\eta =10$ meV is not necessary because the Kubo formula can be calculated even with $\eta =0$ eV. However, we have chosen this value because we got a suitable sharpness of peaks in plots of our simulation. In equation above, ${n}_{\overrightarrow{k},\pm }^{{\rm{FD}},s}=\mathrm{1/1}+exp[({{\mathcal{E}}}_{\overrightarrow{k},\pm }^{s}-\mu )/{k}_{{\rm{B}}}T]$ is the Fermi-Dirac distribution function at the chemical potential $\mu$. As usual ${k}_{{\rm{B}}}$ being the Boltzmann constant and $T$, the absolute temperature.

## Propagation of electromagnetic waves through the monolayer phosphorene

To present the optical activity of phosphorene, we formulate the reflected and transmitted electromagnetic waves of an incident wave in terms of IOC tensor ${\sigma }_{\alpha \beta }$ given by Eq. (12). In our formulation, the incident and scattered plane waves are perpendicular to the phosphorene and the properties of the waves such as intensity, phase and polarization are obtained from the complex two-component electric fields ${\overrightarrow{{\mathcal{E}}}}^{{\rm{i}}}$, ${\overrightarrow{{\mathcal{E}}}}^{{\rm{r}}}=\hat{r}{\overrightarrow{{\mathcal{E}}}}^{{\rm{i}}}$, ${\overrightarrow{{\mathcal{E}}}}^{{\rm{t}}}=\hat{t}{\overrightarrow{{\mathcal{E}}}}^{{\rm{i}}}$ at $z=0$ corresponding to the incident, reflected, and transmitted parts of the electromagnetic waves, respectively. The coefficients $\hat{r}$ and $\hat{t}$ are 2 × 2 complex matrices in $x$-$y$ plane. In the present work, we assume that the thickness of phosphorene is low enough compared to the wavelength of the electromagnetic field, leading to the following boundary conditions for the electric and magnetic fields of the wave:

13a $${\overrightarrow{{\mathcal{E}}}}^{1}={\overrightarrow{{\mathcal{E}}}}^{2},$$

13b $$-\,i{\hat{\tau }}_{y}({\overrightarrow{H}}^{1}-{\overrightarrow{H}}^{2})=\frac{4\pi }{c}\hat{\sigma }\overrightarrow{{\mathcal{E}}},$$

where $c$ and ${\hat{\tau }}_{y}$ are the velocity of light and $y$ component of the Pauli matrices (acts on the real space), respectively. 1(2) denotes the top (bottom) surface of phosphorene. And for the $\hat{\sigma }$ introduced above, we deal with a matrix 2 × 2 including ${\sigma }_{xx/yy}$ diagonal and ${\sigma }_{xy/yx}$ off-diagonal terms, which as it is written below Eq. (12), the Hall conductivities (off-diagonal terms) are zero because of the structure symmetry of phosphorene. For this reason, the reflected and transmitted electromagnetic waves are given only in terms of diagonal terms in the following. Since only the top surface is subjected to the incident wave, ${\overrightarrow{{\mathcal{E}}}}^{1}={\overrightarrow{{\mathcal{E}}}}^{{\rm{i}}}+{\overrightarrow{{\mathcal{E}}}}^{{\rm{r}}}$ and ${\overrightarrow{{\mathcal{E}}}}^{2}={\overrightarrow{{\mathcal{E}}}}^{{\rm{t}}}$ and the magnetic fields for the vacuum at each side are described by the relation $\overrightarrow{H}=\hat{z}\times \overrightarrow{{\mathcal{E}}}$. Thus, the reflection $\hat{r}$ and transmission $\hat{t}$ matrices are given by

14a $$\hat{r}=\left(\begin{array}{cc}-\,\frac{2\pi {\sigma }_{xx}}{2\pi {\sigma }_{xx}+c} & 0\\ 0 & -\,\frac{2\pi {\sigma }_{yy}}{2\pi {\sigma }_{yy}+c}\end{array}\right),$$

14b $$\hat{t}=\left(\begin{array}{cc}\frac{c}{2\pi {\sigma }_{xx}+c} & 0\\ 0 & \frac{c}{2\pi {\sigma }_{yy}+c}\end{array}\right).$$

For a right-circularly polarized electromagnetic wave, the incident elliptically polarized reflected and transmitted electric fields are described by, respectively,

15a $${\overrightarrow{{\mathcal{E}}}}^{{\rm{i}}}=\frac{{{\mathcal{E}}}_{0}}{\sqrt{2}}[\hat{x}+{\mathtt{i}}\hat{y}],$$

15b $${\overrightarrow{{\mathcal{E}}}}^{{\rm{r}}}=\frac{{{\mathcal{E}}}_{0}}{\sqrt{2}}[{r}_{xx}\hat{x}+{\mathtt{i}}{r}_{yy}\hat{y}],$$

15c $${\overrightarrow{{\mathcal{E}}}}^{{\rm{t}}}=\frac{{{\mathcal{E}}}_{0}}{\sqrt{2}}[{t}_{xx}\hat{x}+{\mathtt{i}}{t}_{yy}\hat{y}],$$

in which ${r}_{xx}=-\,2\pi {\sigma }_{xx}/(2\pi {\sigma }_{xx}+c)$, ${r}_{yy}=-\,2\pi {\sigma }_{yy}/(2\pi {\sigma }_{yy}+c)$, ${t}_{xx}=c/(2\pi {\sigma }_{xx}+c)$, and ${t}_{yy}=c/(2\pi {\sigma }_{yy}+c)$. In general, according to the circular polarization ${\hat{\varepsilon }}_{\pm }=(\hat{x}\pm {\mathtt{i}}\hat{y})/2$ for right (+) and left (−) handed circularly polarizations^61^, the general elliptically polarized wave is given by $\overrightarrow{{\mathcal{E}}}={{\mathcal{E}}}_{+}{\hat{\varepsilon }}_{+}+{{\mathcal{E}}}_{-}{\hat{\varepsilon }}_{-}$. This notation helps us to describe the optical activity of phosphorene more conveniently.

As known, the eccentricity (as the measure of ellipticity) and the rotation angle of the polarization ellipse can be connected to the components $${{\mathcal{E}}}_{+}$$ and $${{\mathcal{E}}}_{-}$$ as $${{\mathtt{e}}}_{p}=2/(\sqrt{|{{\mathcal{E}}}_{+}/{{\mathcal{E}}}_{-}|}$$$$+\sqrt{|{{\mathcal{E}}}_{-}/{{\mathcal{E}}}_{+}|})$$ and $${\alpha }_{p}=[\arctan ({\rm{Im}}\,{{\mathcal{E}}}_{+}/{\rm{Re}}\,{{\mathcal{E}}}_{+})$$$$-\arctan ({\rm{Im}}\,{{\mathcal{E}}}_{-}/{\rm{Re}}\,{{\mathcal{E}}}_{-})]/2$$, leading to the following relations in term of the reflection and transmission components:

16a $$0 < {{\mathtt{e}}}_{p}^{r}=\frac{2}{\sqrt{|{\tilde{r}}^{-1}|}+\sqrt{|\tilde{r}|}} < 1,$$

16b $${\alpha }_{p}^{r}=\frac{1}{2}\,\arctan \,\left[\frac{{\rm{Im}}\,\tilde{r}}{{\rm{Re}}\,\tilde{r}}\right],$$

16c $$0 < {{\mathtt{e}}}_{p}^{t}=\frac{2}{\sqrt{|{\tilde{t}}^{-1}|}+\sqrt{|\tilde{t}|}} < 1,$$

16d $${\alpha }_{p}^{t}=\frac{1}{2}\,\arctan \,\left[\frac{{\rm{Im}}\,\tilde{t}}{{\rm{Re}}\,\tilde{t}}\right].$$

with the definition of

17a $$\tilde{r}=\frac{{r}_{xx}+{r}_{yy}}{{r}_{xx}-{r}_{yy}},$$

17b $$\tilde{t}=\frac{{t}_{xx}+{t}_{yy}}{{t}_{xx}-{t}_{yy}},$$

which lead to the non-zero values because of the different IOC components obtained before stemming from the inherent anisotropy structure of phosphorene. From Eqs. (16a) and (16c) one can understand that two limiting values 0 and 1 (as the constraint have to hold) are corresponding to the states of the circular and linear polarization of reflected and transmitted waves, respectively. However, Eqs. (16b) and (16d) refer to the amount of the ellipse axis rotation with respect to the $x$-direction. As the final quantity for the optical activity, we would like to provide some information about the positive absorbed wave intensity originating from the second law of thermodynamics revealing the power dissipation,

18 $$\frac{{I}_{{\mathtt{abs}}}}{{{\mathcal{E}}}_{0}^{2}}=\sum _{\alpha =\{x,y\}}\,\frac{2\pi c\,{\rm{Re}}\,{\sigma }_{\alpha \alpha }}{{(c+2\pi {\rm{Re}}{\sigma }_{\alpha \alpha })}^{2}+4{\pi }^{2}{({\rm{Im}}{\sigma }_{\alpha \alpha })}^{2}}.$$

## Discussion

**Figure 3:**
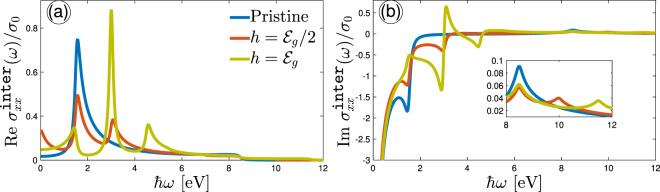
Real (**a**) and imaginary (**b**) parts of the IOC component ${\sigma }_{xx}^{{\mathtt{inter}}}(\omega )$, scaled by ${\sigma }_{0}$ as a function of optical energy for different Zeeman splitting strengths. The temperature is fixed at 10 K in this figure. In the absence of the Zeeman field, only one transition occurs at $\hslash \omega \simeq {{\mathcal{E}}}_{g}$, while four types of transitions emerge at different frequencies depending on the Zeeman field strength. The inside panel in (**b**) illustrates the close-ups of the large limit of frequency.

**Figure 4:**
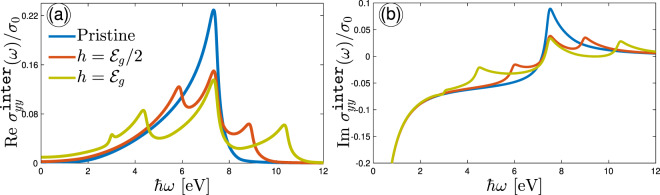
Real (**a**) and imaginary (**b**) parts of the IOC component ${\sigma }_{yy}^{{\mathtt{inter}}}(\omega )$, scaled by ${\sigma }_{0}$ as a function of optical energy for different Zeeman splitting strengths. The temperature is fixed at 10 K. In the absence of Zeeman field, only one transition occurs at $\hslash \omega \simeq 5{{\mathcal{E}}}_{g}$, while four types of transitions emerge at different frequencies depending on the Zeeman field strength.

**Figure 5:**
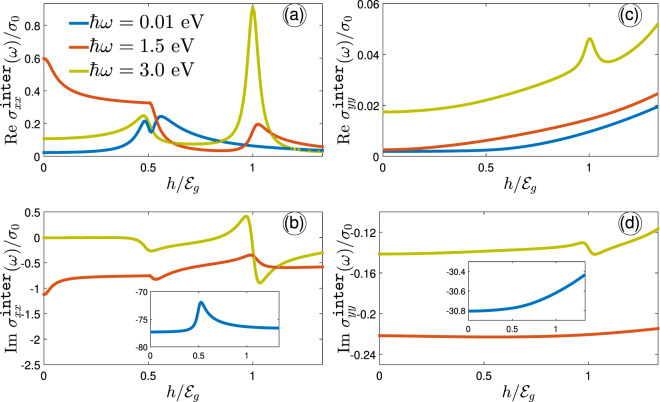
The interband optical conductivity along both armchair {(**a**,**b**)} and zigzag {(**c**,**d**)} directions as a function of $h/{{\mathcal{E}}}_{g}$ at 10 K for different values of the optical energy $\hslash \omega  < {{\mathcal{E}}}_{g}$, $\hslash \omega \simeq {{\mathcal{E}}}_{g}$, and $\hslash \omega  > {{\mathcal{E}}}_{g}$.

We begin with some general remarks, before presenting the results, to our simulations. We analyze the obtained numerical results and discuss the main points supporting the novelty of the present paper by dividing the present section into two parts: (i) optical conductivity and (ii) optical activity. In the first part, first, the temperature is fixed at $T=10$ K and the spin-splitting effects are studied on the IOC as a function of the optical energy $\hslash \omega$ and also the magnetic field $h$ along both armchair and zigzag directions in Figs. 3, 4 and 5. Note that all physical constants $g$, ${\mu }_{{\rm{B}}}$, ${k}_{{\rm{B}}}$, ${m}_{{\rm{e}}}$, and $c$ set to 1 in the present paper. On the other hand, in the optical activity part, a perpendicular circular electromagnetic field is applied to the phosphorene in order to study the ellipticity and the rotation angle of the ellipse polarization. Also, the reflected/transmitted properties and absorption intensity of phosphorene are investigated. We computed conductivities by making use of the rectangle FBZ. For the sake of clarity, we change the name of bands to ${{\mathcal{E}}}_{\overrightarrow{k},-}^{\downarrow }=1$, ${{\mathcal{E}}}_{\overrightarrow{k},-}^{\uparrow }=2$, ${{\mathcal{E}}}_{\overrightarrow{k},+}^{\uparrow }=3$, and ${{\mathcal{E}}}_{\overrightarrow{k},+}^{\downarrow }=4$ hereafter. Finally, we need to introduce the maximum frequency of simulation, ${\omega }_{{\rm{\max }}}$, which in general is completely arbitrary. Unless noted otherwise we take $\hslash {\omega }_{{\rm{\max }}}\simeq 12$ eV to avoid misjudging situations where no anisotropic behavior occurs later.

Before going further, we would like to phrase a comment on the effect of the Fermi energy level on the presented results. In the present work, we consider a constant Fermi energy (${{\mathcal{E}}}_{{\rm{F}}}=0$) at zero and non-zero temperatures. However, the main difference in the presence of the temperature-dependent ${{\mathcal{E}}}_{{\rm{F}}}$ or a finite doping (which also leads to a non-zero Fermi energy) stems from the mutations of the lower-lying energy bands around the ${{\mathcal{E}}}_{{\rm{F}}}$ and eventually the changes in the selection rules of transitions between bands when the optical transitions appear. As soon as the Fermi energy becomes larger than the Zeeman and optical fields, all transitions at low, intermediate, and high regions between bands will be influenced significantly depending on the rate of largeness, leading to new selection rules for the absorption and scattering mechanisms. From these points, in order to have a reliable regime of the energy scales in the experiment, we will evaluate the first case in the following.

Here, one needs to mention that the temperature dependence of the IOC is not needed to be addressed because the band gap of phosphorene 1.52 eV is much larger than the thermal energy ${k}_{{\rm{B}}}T$, leading to almost no change in IOC. For example, for the room temperature 300 K, the above-mentioned thermal energy corresponds to about $26$ meV, which obviously is much smaller than 1.52 eV. On the other hand, this can be analytically checked with the help of Eq. (12), in which the temperature effect is inside of the Fermi-Dirac distribution function difference ${n}_{\overrightarrow{k},+}^{{\rm{FD}}}-{n}_{\overrightarrow{k},-}^{{\rm{FD}}}$ and from Eq. (5), the conduction band minimum (CBM) energy and the valence band maximum (VMB) energy of of 0.34 eV and −1.18 eV, respectively, are obtained at $h=0$ eV, leading to temperature-independent ${n}_{k=0}^{{\rm{FD}}}({\rm{CBM}})-{n}_{k=0}^{{\rm{FD}}}({\rm{VBM}})=1$.

### Optical conductivity

Because the longitudinal optical conductivity relates to the spin-dependent energy difference ${{\mathcal{E}}}_{\overrightarrow{k},+}^{s}-{{\mathcal{E}}}_{\overrightarrow{k},-}^{s}$ between valence and conduction bands, which appears also inside the term ${n}_{\overrightarrow{k},+}^{{\rm{FD}},s}-{n}_{\overrightarrow{k},-}^{{\rm{FD}},s}$, let us begin with the low-energy band structure of phosphorene consisting of two and four bands in the absence and presence of Zeeman splitting field $h$, respectively, as presented in Fig. 2. We mentioned that we are dealing with an undoped four-band phosphorene first at zero temperature so that there are two sets of four types of interband transitions between 1, 2, 3, and 4. The first set includes four the same transitions ${{\mathcal{E}}}_{\overrightarrow{k},-}^{\uparrow ,\downarrow }\mapsto {{\mathcal{E}}}_{\overrightarrow{k},+}^{\uparrow ,\downarrow }$ independent of the direction when $h=0$ eV, leading to the spin degeneracy of bands, whereas the second set of interband transitions refer to the non-zero Zeeman fields. In the latter, at $h={{\mathcal{E}}}_{g}/2$, the first and second types of transitions occur between the energy bands $1\mapsto 3$ and $2\mapsto 4$ equally because of the same splitting gap (equal to ${{\mathcal{E}}}_{g}$) between them (see panels (c) and (d) of Fig. 2). However, the two other ones occur between the bands $1\mapsto 4$ and $2\mapsto 3$ corresponding to the largest (2${{\mathcal{E}}}_{g}$) and smallest (zero) gaps, respectively (see panels (c) and (d) of Fig. 2). As for $h={{\mathcal{E}}}_{g}$, the first and second types of transitions are the same but for the frequencies equal to 2${{\mathcal{E}}}_{g}$, while the two other ones correspond to the largest (3${{\mathcal{E}}}_{g}$) and smallest (${{\mathcal{E}}}_{g}$) gaps, respectively (see panels (e) and (f) of Fig. 2).

It is necessary to mention that for $\mu =0$ eV and $T=0$ K, two energy bands 1 and 2 are occupied, while two other bands 3 and 4 are unoccupied. From this point, at zero temperature and for the undoped case, the transitions $1\mapsto 4$ and $2\mapsto 3$ are forbidden. In order to include these interband optical transitions, we set the temperature to a finite value in the following. For the optical energy we identified three regimes depending on the relation to the band gap of MBP, the beneath band (<${{\mathcal{E}}}_{g}$), the band ($\simeq \,{{\mathcal{E}}}_{g}$) and the above band (>${{\mathcal{E}}}_{g}$) regime. In agreement with our expectation, we find almost zero conductivities for all beneath the band gap optical energies.

Figure 3 shows the real (a) and imaginary (b) parts of IOC of phosphorene along the $x$-direction (scaled by ${\sigma }_{0}$) at $T=10$ K in the presence of magnetic field $h$. The only purpose of $\hslash \omega$ is to be large enough so that interband transitions can be ruled out. In Fig. 3(a), in the absence of the magnetic field, i.e. $h=0$ eV, the real part of ${\sigma }_{xx}^{{\mathtt{inter}}}(\omega )/{\sigma }_{0}$ is a small constant value close to the zero and only one absorption peak at $\hslash \omega \simeq {{\mathcal{E}}}_{g}$ corresponding to the transition ${{\mathcal{E}}}_{\overrightarrow{k},-}^{\uparrow ,\downarrow }\mapsto {{\mathcal{E}}}_{\overrightarrow{k},+}^{\uparrow ,\downarrow }$ appears because spin-splitting is absent, but for higher values of the Zeeman field the spin-dependent dissipation channel corresponding to the transitions $2\mapsto 3$, $\{1\mapsto 3,2\mapsto 4\}$, and $1\mapsto 4$ takes the role in the IOC. Interestingly, for $h={{\mathcal{E}}}_{g}/2$, the absorption peaks (the peaks in the real part) corresponding to these transitions take place at $\hslash \omega \simeq 0$, 1.5, and 3 eV, respectively. The first frequency is for the gapless phase (according to the panels (c) and (d) of Fig. 2), while the second and third frequencies are proportional to the gapped phases between spin bands. However, as the Zeeman field is increased further, i.e. $h={{\mathcal{E}}}_{g}$, the sort of absorption peaks is changed due to the new Dirac-like points between $\Gamma$ and *X*/*Y* points. In this case, three absorption peaks at frequencies $\hslash \omega \simeq 1.5$, 3, and 4.5 eV appear corresponding to the transitions between the bands mentioned above, respectively. These types of transitions are all for the gapped phase (according to the panels (e) and (f) of Fig. 2).

As for the imaginary part of IOC along the $x$-direction, i.e. Figure 3(b), peaks describe by the optical field-induced scattering of electrons. Because of the Kramers-Kroning relation between the real and imaginary part of ${\sigma }_{xx}^{{\mathtt{inter}}}(\omega )/{\sigma }_{0}$, the position of scattering peaks (in ${\rm{Im}}\,{\sigma }_{xx}^{{\mathtt{inter}}}(\omega )/{\sigma }_{0}$) should be the same with the peak positions of absorption peaks (in ${\rm{Re}}\,{\sigma }_{xx}^{{\mathtt{inter}}}(\omega )/{\sigma }_{0}$), which our results confirm this relation. There is one important phenomenon in Fig. 3(b) that a back-scattering is occurring at $\hslash \omega \simeq 3$ eV because ${\rm{Im}}\,{\sigma }_{xx}^{{\mathtt{inter}}}(\omega )/{\sigma }_{0}$ gets a positive peak at this point, which refers to the new created Dirac-like cone in panel (e) of Fig. 2. Another point refers to the peaks appearing at $\hslash \omega \ge 8.5$ eV. These peaks mean that there are very weak absorptions corresponding to the latest valence and conduction bands, which totally the propagation direction of electrons has been reversed. And at very high frequencies, the conductivity approaches zero because based on our model, there are no more bands corresponding to these optical energies. If one is interested in having more peaks even at high enough optical energies, another band model is required to support them. The results for the case of $h=0$ eV are in good agreement with the work by Yang *et al*.^51^.

Inherent asymmetry property of charge carriers in different directions in phosphorene results in different treatments of IOC along the $y$-direction (zigzag edge), as shown in Fig. 4. It implies that when exploring the ${\sigma }_{yy}^{{\mathtt{inter}}}(\omega )/{\sigma }_{0}$, different selection rules for transition between bands are expected at $T=10$ K. This can be concluded from parameters ${\nu }_{\overrightarrow{k}}^{\alpha }$ and ${\chi }_{\overrightarrow{k}}^{\alpha }$ in the current operator ${J}_{\alpha }$. Because of the different slopes of bands in Fig. 2 for $\Gamma -Y$ directions, the transitions mentioned for the armchair direction change as follows. For $h=0$ eV, again, spin degeneracy is valid and only four the same interband transitions ${{\mathcal{E}}}_{\overrightarrow{k},-}^{\uparrow ,\downarrow }\mapsto {{\mathcal{E}}}_{\overrightarrow{k},+}^{\uparrow ,\downarrow }$ occur, as demonstrated as peaks at $\hslash \omega \simeq 7.5$ eV (5${{\mathcal{E}}}_{g}$) in both real and imaginary parts of ${\sigma }_{yy}^{{\mathtt{inter}}}(\omega )/{\sigma }_{0}$. For $h={{\mathcal{E}}}_{g}/2$, the transitions $2\mapsto 3$, {$1\mapsto 3$, $2\mapsto 4$}, and $1\mapsto 4$ occur at different optical energies compared to the ${\sigma }_{xx}^{{\mathtt{inter}}}(\omega )/{\sigma }_{0}$. Figure 4(a) shows the position of absorption peaks corresponding to these transitions at $\hslash \omega \simeq 4{{\mathcal{E}}}_{g}\mathrm{,5}{{\mathcal{E}}}_{g}$, and $6{{\mathcal{E}}}_{g}$, respectively. On the other hand, the scattering peaks in Fig. 4(b) for $h={{\mathcal{E}}}_{g}/2$ take place at the same positions due to the Kramers-Kroning relation, which provide some back scattering mechanisms for $\hslash \omega \ge 5{{\mathcal{E}}}_{g}$. For the strength $h={{\mathcal{E}}}_{g}$, the peaks corresponding to the transitions between bands $2\mapsto 3$ and $1\mapsto 4$ go away from each other and take place at $\hslash \omega \simeq 3{{\mathcal{E}}}_{g}$ and $7{{\mathcal{E}}}_{g}$, while the peak corresponding to the transitions {$1\mapsto 3$, $2\mapsto 4$} do not change. Due to the gap closure (Dirac-like cone) between the points $\Gamma$ and $Y$, for $h={{\mathcal{E}}}_{g}$, there is one peak at low frequencies corresponding to the transitions at the cross point in which the bands 2 and 3 are deformed. Similar to the previous cases, the behavior of the Im ${\sigma }_{yy}^{{\mathtt{inter}}}(\omega )/{\sigma }_{0}$ is the same for $\hslash \omega \ge 5{{\mathcal{E}}}_{g}$, i.e. it can be positive, as indicated in Fig. 4(b).

In order to further substantiate the findings above we plot the IOC along both armchair and zigzag directions as a function of $h$ for different values of the optical energy $\hslash \omega  < {{\mathcal{E}}}_{g}$, $\hslash \omega \simeq {{\mathcal{E}}}_{g}$, and $\hslash \omega  > {{\mathcal{E}}}_{g}$ in Fig. 5. For obvious reasons, we needed to restrict the applicability of our studies to the finite optical energy regions, i.e. not high values of frequency, which provide convenient results. Also, according to Fig. 2, we divide the analysis of results for spin bands into three phases: (i) gapped phase for both spins, (ii) gapless and gapped phases and (iii) the Dirac-like phase crossing spin-up and spin-down.

In the gapped phase for both spins at $h=0$ eV, the real part of both conductivities ${\sigma }_{xx}^{{\rm{inter}}}(\omega )/{\sigma }_{0}$ and ${\sigma }_{yy}^{{\rm{inter}}}(\omega )/{\sigma }_{0}$ are almost zero for $\hslash \omega  < {{\mathcal{E}}}_{g}$ and increase as the optical energy reaches to $\hslash \omega \simeq {{\mathcal{E}}}_{g}$. Furthermore, when the optical energy comes to the $\hslash \omega  > {{\mathcal{E}}}_{g}$, the conductivity along the armchair direction decreases, while the one along the zigzag direction continues to increase. Now, let us turn to the second case; for $\hslash \omega  < {{\mathcal{E}}}_{g}$ it has two peaks and one dip around the point $h={{\mathcal{E}}}_{g}/2$ along the armchair edge, whereas it increases a little bit with the Zeeman splitting field along the zigzag edge stemming from the intrinsic highly anisotropy in phophorene. Moreover, at higher optical frequencies in which $\hslash \omega \simeq {{\mathcal{E}}}_{g}$ and $\hslash \omega  > {{\mathcal{E}}}_{g}$ the IOC along the $x$-direction increases first and then decreases, whereas IOC increases slightly along the $y$-direction with $\hslash \omega$ at $h={{\mathcal{E}}}_{g}/2$. Unlike the previous phase this happens only for the absorption spectra and the scattering spectra increases with $\hslash \omega$ at $h={{\mathcal{E}}}_{g}/2$ (see Fig. 5(b,d)). And as for the third phases, i.e. the Dirac-like phase at $h={{\mathcal{E}}}_{g}$, the dissipative real conductivities increase with $\hslash \omega$ (see Fig. 5(a,c)) and take the maximum intensity at higher frequencies ($\hslash \omega  > {{\mathcal{E}}}_{g}$). In this case, also the behaviors of the imaginary conductivities are the same as the real ones, i.e. both imaginary $x$- and $y$-direction-dependent conductivities increase with $\hslash \omega$ at $h={{\mathcal{E}}}_{g}$.

In general, the rate of changes in $x$- and $y$-directions is completely different with a big factor. From the analysis above, we note that the anisotropic treatments of Re${\sigma }_{xx}^{{\rm{inter}}}(\omega )/{\sigma }_{0}$ and Im${\sigma }_{xx}^{{\rm{inter}}}(\omega )/{\sigma }_{0}$ with the corresponding ones in $y$-direction take place in the vicinity of transition points when the optical energy is equal to the band gap of the system (${{\mathcal{E}}}_{g}\simeq 1.52$ eV). When $\hslash \omega$ becomes more and/or less than ${{\mathcal{E}}}_{g}$, the conductivities show some irregularities in their values. Another strong anisotropy property between Re${\sigma }_{xx}^{{\rm{inter}}}(\omega )/{\sigma }_{0}$ and Re${\sigma }_{yy}^{{\rm{inter}}}(\omega )/{\sigma }_{0}$ refers to the value of conductivity at very small and very large fields *h*. Conductivities vanish at very small fields in both cases, while Re${\sigma }_{xx}^{{\rm{inter}}}(\omega )/{\sigma }_{0}$ (Re${\sigma }_{yy}^{{\rm{inter}}}(\omega )/{\sigma }_{0}$) approaches zero (increases) at very large fields, affecting the scattering processes in the corresponding imaginary parts.

### Optical activity

In this part, we peruse the Zeeman spin-splitting effects on the optical activity of phosphorene in the presence of a perpendicular circularly polarized light formulated in Sec. 4 for the eccentricity e_*p*_, the phase shift *α*_*p*_, and the absorption intensity ${I}_{{\mathtt{abs}}}/{{\mathcal{E}}}_{0}^{2}$ of reflected and transmitted electromagnetic waves. As the first remark at this part, we note that due to the relation ${\sigma }_{xx}\ne {\sigma }_{yy}$ in phosphorene, which is a direct consequence of previous part the reflected and transmitted waves are elliptic in general, not the circular one like the incident wave. Then, we have three regimes of the polarization including circular, elliptic, and linear polarizations.

**Figure 6:**
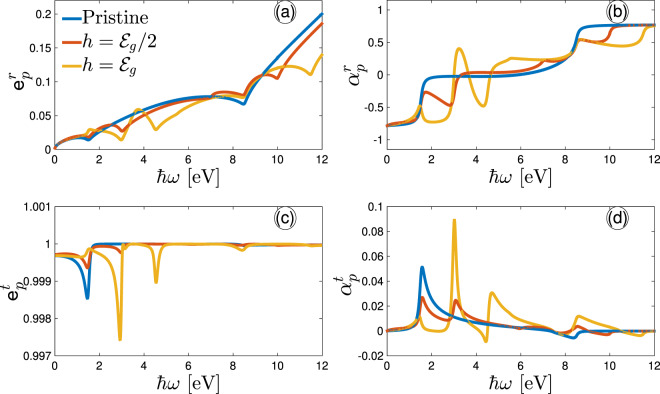
Optical frequency- and Zeeman spin-splitting-dependent {(**a**,**c**)} eccentricity and {(**b**,**d**)} phase shift of the reflected and transmitted electromagnetic wave at $T=10$ K.

Figure 6{(a–d)} demonstrate the reflected and transmitted properties, respectively, as a function of optical frequency $\hslash \omega$ at different Zeeman strengths and temperature $T=10$ K. First of all, we analyse the reflected electromagnetic waves in panels (a) and (b). The curves in (a) show that the reflected light is almost elliptic in a wide range if the frequency at both weak and strong Zeeman fields with no difference below the energy band gap ${{\mathcal{E}}}_{g}$. For comparison, the dips and peaks of ${{\mathtt{e}}}_{p}^{r}$ for different Zeeman strengths show the different kinds of polarization corresponding to certain optical frequencies of interband transitions, which are going to be elliptic ones by taking the values larger than zero. As expected, since both components of the conductivity become isotropic at $\hslash \omega  > 8.5$ eV the same polarization for different Zeeman fields can be seen. In short, in this case, no linear polarization takes place. Next, we study the ellipse rotation angles ${\alpha }_{p}^{r}$ for the reflected electromagnetic waves in Fig. 6(b). The most interesting situation occurs for different optical frequencies, well starting different types of transitions provided before. For $\hslash \omega \simeq 5{{\mathcal{E}}}_{g}$ at $h=0$ eV the ellipses are strongly rotated and show the same but opposite treatments for $\hslash \omega  > 5{{\mathcal{E}}}_{g}$ as the case of $\hslash \omega  < {{\mathcal{E}}}_{g}$. However, the position of ellipse rotations is different when the Zeeman field is increased stemming from different types of interband transitions than the zero Zeeman field. For instance, at $h={{\mathcal{E}}}_{g}/2$, ellipse rotation takes place at $\hslash \omega \simeq 2{{\mathcal{E}}}_{g}$, whereas it appears at $\hslash \omega \simeq 2{{\mathcal{E}}}_{g}$ and $3{{\mathcal{E}}}_{g}$ when $h={{\mathcal{E}}}_{g}$. From Fig. 6(b), one finds that the ellipse rotation angle ${\alpha }_{p}^{r}$ remains unchanged and independent of the Zeeman field strength for high enough frequencies.

As for the transmitted waves, as depicted in Fig. 6(c), ${{\mathtt{e}}}_{p}^{t}$ shows few tiny peaks at which the eccentricity reaches to 1 corresponding to the linear polarization in a wide range of $\hslash \omega$ for all Zeeman field strengths. This is a surprising result which reports that most of the light transmits into the phosphorene lattice and a bit percentage of the wave is reflected, introducing and/or confirming phosphorene as the best candidate in the nano-optoelectronic semiconductor industry. In comparison with panel (b), less ellipse rotation angels ${\alpha }_{p}^{t}$ appear for the transmitted wave and only at strong enough Zeeman fields ($h={{\mathcal{E}}}_{g}$), a rotation occurs at $\hslash \omega \simeq 3{{\mathcal{E}}}_{g}$.

**Figure 7:**
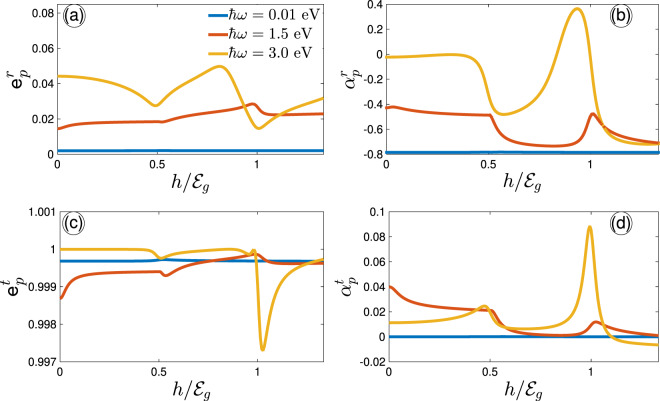
Eccentricity (**a**) and phase shift (**b**) of the reflected light as a function of the Zeeman splitting field at low and intermediate optical frequencies at $T=10$ K. The corresponding plots for the transmitted light is plotted in (**c** and **d**), respectively.

In order to show more deeply how the Zeeman spin-splitting field affects the polarization of the incident light after reflecting from or transmitting into the system at low and intermediate optical frequencies, we plot ${{\mathtt{e}}}_{p}^{r}$, ${\alpha }_{p}^{r}$, ${{\mathtt{e}}}_{p}^{t}$, and ${\alpha }_{p}^{t}$ in Fig. 7. First, we can see that these quantities are almost constant about the zero optical frequency, i.e. linear blue curves in Fig. 7(a–d). Second, all the quantities data at intermediate frequencies present the highest peaks/dips in comparison with the lower optical energies ($\hslash \omega \simeq {{\mathcal{E}}}_{g}$ eV). In all panels, the data approximately approach a constant value at strong enough Zeeman fields originating from the isotropic conductivity components. Analysis of the effect of the Zeeman field on the eccentricity of reflected and transmitted waves in Fig. 7(a,c), respectively, extracts elliptic and almost linear polarizations at all. While the corresponding phase shifts in Fig. 7(b,d) depict a rotation of reflected light at $h=1.25$ eV at $\hslash \omega \simeq {{\mathcal{E}}}_{g}$, no rotation is occurred for the transmitted waves. Certainly, this should be reasonable since Fig. 6 generally confirms the same behaviors. As the Zeeman field becomes strong, both reflected and transmitted waves do not change significantly, clearly representing that the phosphorene system is suppressed to involve with the strong scattering process, which can be observed in Fig. 5.

**Figure 8:**
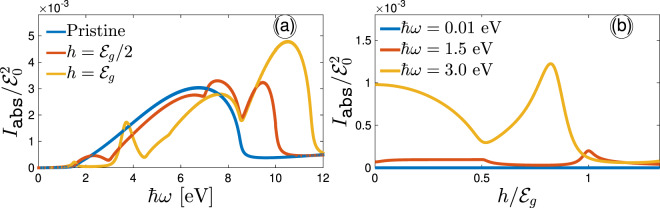
The intensity of absorbed light as a function of (**a**) incident optical frequency and (**b**) the Zeeman splitting effects at different Zeeman fields and optical frequencies, respectively, at $T=10$ K.

We now turn to the results of our intensity of absorbed light of phosphorene calculations to gain insights into the role of Zeeman splitting and optical frequency on ${I}_{{\mathtt{abs}}}/{{\mathcal{E}}}_{0}^{2}$ in Fig. 8, which is strongly dependent on the components of the conductivity $\sigma$. ${I}_{{\mathtt{abs}}}/{{\mathcal{E}}}_{0}^{2}$ should always be positive due to the fact that Re${\sigma }_{\alpha \alpha }^{{\mathtt{inter}}}(\omega )/{\sigma }_{0}$, as shown in Fig. 3(a) and 4(a) is positive forever. In Fig. 8(a), for the optical energies below the band gap region between valence and conduction states, independent of the Zeeman field strength, the phosphorene is almost *transparent* and ${I}_{{\mathtt{abs}}}/{{\mathcal{E}}}_{0}^{2}\simeq 0$ due to the absence of charge carriers, in agreement with refs. ^62,63^. Beyond the band gap, which both conductivities exhibits finite values, arising from charge excitations and transitions, ${I}_{{\mathtt{abs}}}/{{\mathcal{E}}}_{0}^{2}$ increases for all Zeeman fields and oscillates depending on $h$ amount (due to the phase transitions mentioned in Fig. 1) and eventually an absorption up to less than 1% is possible. This 1% of absorption is neglectable and one can conclude that the phosphorene is almost transparent at low and intermediate regions of the Zeeman splitting, but independent of the incident light frequency $\hslash \omega$. This originates from Fig. 6(c) and 7(c) of which the light is almost transmitted from the system. Moreover, due to the isotropic conductivities ${\sigma }_{xx}^{{\mathtt{inter}}}(\omega )/{\sigma }_{0}$ and ${\sigma }_{yy}^{{\mathtt{inter}}}(\omega )/{\sigma }_{0}$ at large frequencies, ${I}_{{\mathtt{abs}}}/{{\mathcal{E}}}_{0}^{2}$ exhibits the same plateaus, as represented in Fig. 8(a).

To more deeply clarify the Zeeman field effects at certain frequencies, ${I}_{{\mathtt{abs}}}/{{\mathcal{E}}}_{0}^{2}$ as a function of $h$ at low and intermediate frequencies are more obvious in Fig. 8(b). The zero plateau is a straight consequence of full transparency of phosphorene when $\hslash \omega  < {{\mathcal{E}}}_{g}$. On the other hand, the prominent peaks and non-zero $${I}_{\begin{array}{c}{\mathtt{abs}}\end{array}}/{{\mathcal{E}}}_{0}^{2}$$ gradually arise when $\hslash \omega \simeq {{\mathcal{E}}}_{g}$ with a further increase in ${I}_{{\mathtt{abs}}}/{{\mathcal{E}}}_{0}^{2}$ when $\hslash \omega  > {{\mathcal{E}}}_{g}$. As expected, it implies that the Zeeman field is affecting the absorption and scattering processes. The $\hslash \omega \simeq {{\mathcal{E}}}_{g}$-induced peak and height of finite intensity of absorbed light change drastically and also a dip emerges at $h={{\mathcal{E}}}_{g}/2$ due to the gapless phase between valence and conduction bands. Nevertheless, mainly due to the backscattering effect occurring when $h$ is increased, no sharp change is expected for ${I}_{{\mathtt{abs}}}/{{\mathcal{E}}}_{0}^{2}$ curves, as confirmed in Fig. 8(b).

## Conclusions

We gained several insights on the optoelectronic properties of phosphorene, especially in applicability proposing a Zeeman spin-splitting field. Applying the tight-binding Hamiltonian model for hopping parameters up to fifth terms between nearest-neighbors phosphorus atoms, we found the energy dispersions of the model. By construction, we can separate the spin-up and spin-down bands when it comes to investigating their respective behavior in determining new electronic phases of the system. Building on this field, we have introduced three regimes to the system. For the case of the absence of the Zeeman field, two the same gapped phases for both spins are evident. For stronger fields, gapless and gapped phases are observed. By contrast, if the Zeeman fields are strong enough, the band inversion emerges and the spin-up bands confront a Dirac-like cone phase, while spin-down ones still suffer from a gapped phase. These are along both armchair and zigzag directions with almost linear and parabolic dispersions, respectively.

Then using the Kubo formalism, assuming large enough energy of the incident light, we obtained an analytical description for the interband optical conductivity (IOC) from which we further derived the relations of eccentricity and shift phase of reflected and transmitted electromagnetic waves of an incident wave. Exciting the particles in the presence of the Zeeman field was to derive different types of interband transitions along both armchair and zigzag directions due to the separated spin bands. Whenever the Zeeman field was weak we found more absorption and scattering mechanism at low and intermediate optical energy ranges compared to other frequencies. This stems from the lack of the valence and conduction bands proportional to the high enough frequencies. For the sake of clarity, we characterized the direction-dependent IOC behaviors as a function Zeeman field by considering three regimes of the optical energy of which the model provides convenient results. Eventually, we ended up to an irregular (regular) treatment for IOC along the armchair (zigzag) direction, respectively.

Not only, as we already mentioned, we obtain the Zeeman field- and frequency-dependent IOC behavior along both directions but also we identified the propagation of electromagnetic waves of an incident circularly right-handed polarized light through phosphorene. Therefore, we focused on the eccentricity (to gain the circular, elliptical and linear polarizations) and the shift phase (to gain the ellipse rotation angle of polarization) of the reflected and transmitted electromagnetic waves. A further investigation as part of the current research provided new insights into the positive absorbed wave intensity of phosphorene. In agreement with our expectation, we found eccentricity of transmitted waves of about 1 meaning that the phosphorene is almost transparent inherently. Thus, the obvious dependence of IOC and optical activity of phosphorene on the Zeeman field and frequency might be fruitful to hold rich perspectives in the design of optoelectronic applications. As an outlook, the most promising ansatz to close our model to the logic devices involves the addition of indisputable impairments to the system in hopes of finding new insights.

## Data Availability

The data that support the findings of this study are available from the corresponding author upon reasonable request.
